# “Prone positioning as an emerging tool in the care provided to patients infected with COVID-19: a scoping review”

**DOI:** 10.1590/1518-8345.5556.3501

**Published:** 2021-11-19

**Authors:** Layla Alba de Matias, Eugenio Esmeraldino Mendes, Aline Oenning Baggio, Chaiana Esmeraldino Mendes Marcon

**Affiliations:** 1 Universidade do Sul de Santa Catarina, Tubarão, SC, Brazil.

Dear Editor, The study by Marília Souto de Araújo and colleagues^(1)^ presents the benefits and risks of applying the prone position in the care process of hospitalized patients with COVID-19, and it has concluded that more positive outcomes stood out from the negative ones, thus showing the reduction of hypoxemia and mortality.

The prone position was already a maneuver used to fight hypoxemia in patients with Acute Respiratory Distress Syndrome, showing improvement by changing the pulmonary regions compressed by the heart, enabling increased cardiac output and reduced lung weight, which is increased by edema and is aggravated by gravity, and may reduce the action of your weight when you are in a prone position. It is important to highlight that its use presents significant improvements for patients in intensive care and in wards, representing a tool that can be established in early treatment, shortening the patient’s length of hospital stay and having positive effects on clinical outcomes^(2)^.

The main complications of the prone position are due to the development of pressure ulcers, brachial plexus injury and difficulties in venous access. For this reason, intensive care requires teams that are prepared to prevent such progress, making changes in the patient’s position to reduce pressure points and avoid nerve damage. The use of pillows and pronation cycles lasting 12 to 16 hours can contribute to the reduction of ulcers, preventing its consequences which are related to high mortality. Furthermore, its occurrence presents a high risk of developing osteomyelitis or sepsis, in addition to potentiating bleeding that is aggravated by the use of anticoagulants in the COVID-19 treatment^(3)^.

The profile of patients who are more likely to aggravate their COVID-19 situation are associated to obesity and inflammation, being considered difficult to manage cases due to their prone position, large body extension, possible edema caused by immobility and risk of deep vein thrombosis of the lower limbs. For this reason, the search for appropriate venous access is necessary, and the use of commonly punctured cervical, thoracic and femoral veins is inadequate. In view of this, the upper extremities remain an option for peripherally inserted central catheters. In addition, the superficial femoral vein can also be considered an adequate access route in the prone position, leaving space in the literature for further clarification regarding its use^(4-5)^.

The prone position is an essential tool for the treatment of COVID-19 hospitalized patients. Its use generates injuries that require attention and training of the health team, preventing the death of patients and possible aggravations that can cause greater wear of professionals and increase the health services costs, which is already overloaded by the pandemic^(1)^. The presentation of solutions for the negative aspects of using the prone position and its requirement prevents greater system overload, using it as effectively as possible in order to assist in the treatment of respiratory syndrome.
